# Oxidized Low-Density Lipoprotein-Deteriorated Psoriasis Is Associated with the Upregulation of Lox-1 Receptor and Il-23 Expression In Vivo and In Vitro

**DOI:** 10.3390/ijms19092610

**Published:** 2018-09-03

**Authors:** Chun-Ming Shih, Chien-Yu Huang, Kuo-Hsien Wang, Chun-Yao Huang, Po-Li Wei, Yu-Jia Chang, Chi-Kun Hsieh, Kuan-Ting Liu, Ai-Wei Lee

**Affiliations:** 1 Department of Internal Medicine, School of Medicine, College of Medicine, Taipei Medical University, Taipei 11031, Taiwan; cmshih53@tmu.edu.tw (C.-M.S.); cyhuang@tmu.edu.tw (C.-Y.H.); faceeric0302@yahoo.com.tw (C.-K.H.); jack841023yes@gmail.com (K.-T.L.); 2 Division of Cardiology and Cardiovascular Research Center, Taipei Medical University Hospital, Taipei 11031, Taiwan; 3 Department of Surgery, School of Medicine, College of Medicine, Taipei Medical University, Taipei 11031, Taiwan; cyh@tmu.edu.tw (C.-Y.H.); poliwei@tmu.edu.tw (P.-L.W.); r5424012@tmu.edu.tw (Y.-J.C.); 4 Division of General Surgery, Department of Surgery, Shuang Ho Hospital, Taipei Medical University, Taipei 11031, Taiwan; 5 Department of Dermatology, Taipei Medical University Hospital, Taipei 11031, Taiwan; 162014@h.tmu.edu.tw; 6 Graduate Institute of Cancer Biology and Drug Discovery, Graduate Institute of Clinical Medicine and Department of Surgery, School of Medicine, College of Medicine, Taipei Medical University, Taipei 11031, Taiwan; 7 Department of Anatomy and Cell Biology, School of Medicine, College of Medicine, Taipei Medical University, Taipei 11031, Taiwan

**Keywords:** oxLDL, LOX-1, psoriasis

## Abstract

Psoriasis is a chronic inflammatory skin disease. Even though scientists predict that abnormalities in lipid metabolism play an important role in the pathogenesis of psoriasis, the actual underlying mechanisms are still unclear. Therefore, understanding the possible relationship between mechanisms of the occurrence of psoriasis and dyslipidemia is an important issue that may lead to the development of effective therapies. Under this principle, we investigated the influences of hyperlipidemia in imiquimod (IMQ)-induced psoriasis-like B6.129S2-*Apoe^tm1Unc^*/J mice and oxidized low-density lipoprotein (oxLDL) in tumor necrosis factor (TNF)-α-stimulated Hacat cells. In our study, we showed that a high-cholesterol diet aggravated psoriasis-like phenomena in IMQ-treated B6.129S2-*Apoe^tm1Unc^*/J mice. In vitro analysis showed that oxLDL increased keratinocyte migration and lectin-type oxLDL receptor 1 (LOX-1) expression. Evidence suggested that interleukin (IL)-23 was a main cytokine in the pathogenesis of psoriasis. High-cholesterol diet aggravated IL-23 expression in IMQ-treated B6.129S2-*Apoe^tm1Unc^*/J mice, and oxLDL induced IL-23 expression mediated by LOX-1 in TNF-α-stimulated Hacat cells. Therefore, it will be interesting to investigate the factors for the oxLDL induction of LOX-1 in psoriasis. LOX-1 receptor expression may be another novel treatment option for psoriasis and might represent the most promising strategy.

## 1. Introduction

Psoriasis is a chronic inflammatory skin disease of unknown etiology that is estimated to affect 2–3% of the general population worldwide [1]. Even though conventional psoriasis has been thought to be a dermatological disease, current medical literature reports that psoriasis is actually chronic inflammation [2]. Psoriasis is characterized by patches of abnormal skin that are typically red, itchy, and scaly. Psoriasis affects the skin, nails, and, sporadically, the joints and is characterized by epidermal hyperproliferation, abnormal differentiation of keratinocytes, and lymphocyte infiltration, which results in the raised plaques that constantly molt scales originating from excessive growth of the epidermal skin layer [3,4]. There are numerous inflammatory cells and mediators of psoriasis. At the site of psoriatic inflammation, activated T cells predominantly release cytokines, such as tumor necrosis factor alpha (TNF-α) and IL-2 [5,6]. TNF-α not only contributes to the hyperproliferation of keratinocytes in the skin but also stimulates adhesion molecule expression on vascular endothelial cells [4].

Previous reports demonstrated that psoriasis is associated with an increased risk of atherosclerotic cardiovascular disease, obesity, and hyperlipidemia that potentially increases morbidity and mortality, which has been suspected for many years [7]. Patients with psoriasis have a higher morbidity rate of cardiovascular diseases [8]. Additionally, arrhythmias are more universal with psoriasis [9]. In another study, it was suggested that psoriasis patients had a thicker intima-media and impaired endothelial function of the common carotid artery [10]. According to a descriptive cohort study, patients with psoriasis had higher risks of myocardial infarction, atherosclerosis, angina, and stroke [11]. The association of psoriasis with metabolic syndrome has recently been extensively established. Metabolic syndrome contains a variety of cardiovascular risk factors, such as atherosclerosis, dyslipidemia, and obesity [12,13,14]. Epidemiological studies have shown a higher incidence of atherosclerosis, dyslipidemia, and obesity in populations with a pathological lipoprotein profile, which includes high levels of total cholesterol, low-density lipoprotein (LDL), triglyceride and low levels of high-density lipoprotein (HDL) [15]. Abnormalities in lipid metabolism play an important role in the pathogenesis of psoriasis [16]. Although lipid metabolism disorder is one of the cardiovascular risk factors, some studies disagree with the role of hyperlipidemia in psoriasis. To date, understanding the possible mechanisms underlying the association between psoriasis and the atherosclerotic lipoprotein profile is important and may lead to the development of effective therapies. During the inflammatory process, oxidative stress generates oxidation, alkylation, and glycation to modify lipoproteins [17] that are recognized by scavenger receptors, such as scavenger receptor (SR-B1), lectin-like oxidized low-density lipoprotein receptor-1 (LOX-1), and scavenger receptor A (SRA), as well as induced activation of the intracellular signaling pathway. Furthermore, scavenger receptors with an important lipoprotein transportation capability have been explored, and their regulation has been demonstrated in vascular endothelial cells, aortic smooth muscle cells, Kupffer cells, and dendritic cells of the liver [18]. However, the function and expression of scavenger receptors still needs to be elucidated, although keratinocytes play critical roles in psoriasis and expressing them had been explored [19]. In other words, the functional basis of the regulatory mechanisms of scavenger receptor expression and their roles in psoriasis are not well established. Thus, the first purpose of this study was to evaluate the effects of modified lipoproteins in psoriasis-like mouse and TNF-α-stimulated keratinocytes. Additionally, the second purpose of this study was to identify the scavenger receptors in psoriasis pathogenesis.

## 2. Results

### 2.1. Topical Imiquimod Cream Treatment Does Not Affect Serum Lipid Levels or Liver or Kidney Function in Hc-Diet-Fed B6.129s2-Apoe^tm1unc^/J Mice

In order to confirm that topical imiquimod (IMQ) treatment does not affect serum lipid levels or liver or kidney function, and high-cholesterol (HC)-diet-fed indeed induces the hyperlipidemia in B6.129S2-Apoe^tm1Unc^/J mice, the blood biochemical analysis were performed. The levels of total-cholesterol, Serum blood urea nitrogen (BUN), creatinine, alanine aminotransferase (ALT), aspartate aminotransferase (AST), triglyceride (TG), and creatinine are shown in Table 1. Before beginning the experiment, the baseline total-cholesterol, TG, ALT, AST, BUN, and creatinine were similar in all the groups. During the experimental period (the end of the 6th week), the levels of all the parameters did not differ significantly in B6.129P2-Apoe^tm1Unc^/J mice with or without IMQ treatment in the normal chow diet. In contrast, mice fed the HC had an increased total-cholesterol level, which reached 1213.5 ± 174.2 mg/dL and 1047.0 ± 198.2 mg/dL in the treatment groups without and with IMQ, respectively. TG reached 92.0 ± 18.4 mg/dL and 94.0 ± 17.7 mg/dL in the treatment groups without and with IMQ, respectively. Mice fed with the HC diet had significantly induced accumulation of total-cholesterol and TG compared to the groups fed the normal chow diet. However, the plasma lipid levels did not increase with the IMQ treatment in HC-diet-fed mice. Additionally, IMQ treatment did not alter the ALT, AST, BUN, and creatinine in the HC-diet fed mice. The results showed that topical IMQ cream did not affect the kidney or liver function or the lipid profile in the HC diet-induced B6.129P2-Apoe^tm1Unc^/J mice.

### 2.2. HC-Diet Aggravates Psoriasis-Like Phenomena in IMQ-Treated B6.129S2-Apoe^tm1Unc^/J Mice

**Figure 1:**
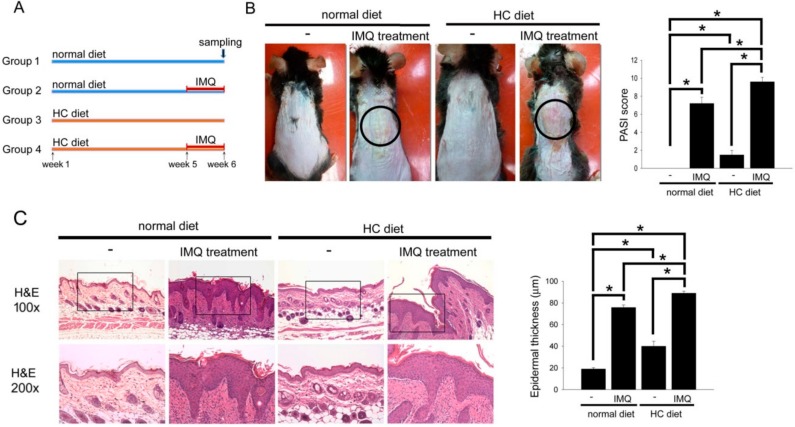
HC-diet aggravated the psoriasis-like phenomena and epidermis hyperplasia in IMQ-treated B6.129S2-Apoe^tm1Unc^/J mice. (**A**) The progress of the experiment and animal groupings are presented. (**B**) The representative photos of the mice that received IMQ treatment in the normal chow diet or HC-diet groups. The PASI score was demonstrated as a bar graph (right). (**C**) Structural feature characteristics of IMQ-induced psoriasis-like skin were analyzed on H&E stained sections, and analysis using microscopy at 100× and 200× magnification of the slide. The epidermis thickness was quantified and presented as a bar graph (right). The results were expressed as the mean ± SD. * *p* < 0.05 was considered statistically significant.

The representative photos (Figure 1B) show the psoriasis-like skin in mice fed the normal chow or HC diet with or without IMQ treatment. Significantly, IMQ treatment induced the occurrence of psoriasis-like skin in normal chow diet-fed mice. More serious psoriasis-like skin and scarring (circle area) were observed in IMQ treatment plus HC-diet-fed mice than that in IMQ treatment plus normal chow diet-fed mice. The quantification of the severity of psoriasis-like skin using the PASI score is shown as a bar graph. IMQ treatment induced the elevation of PASI score in normal chow diet-fed mice. Interestingly, the HC-diet slightly induced the elevation of the PASI score. More serious Psoriasis Area and Severity Index (PASI) score was observed in IMQ treatment plus HC-diet-fed mice than that in IMQ treatment plus normal chow diet-fed mice. Additionally, structural features characteristic of IMQ-induced psoriasis-like skin were analyzed on hematoxylin and eosin (HE)-stained sections. As shown in Figure 1C, normal skin had a thin epidermis. The HC-diet slightly induced the hyperplasia of the epidermis. Furthermore, the mouse skin revealed severe keratinocyte hyperplasia in HC-diet-fed mice for 6 weeks after IMQ treatment. These results suggest that the HC-diet aggravated psoriasis-like phenomena and epidermis hyperplasia in IMQ-treated B6.129S2-Apoe^tm1Unc^/J mice.

### 2.3. OxLDL Increases Keratinocyte Migration and LOX-1 Expression

**Figure 2:**
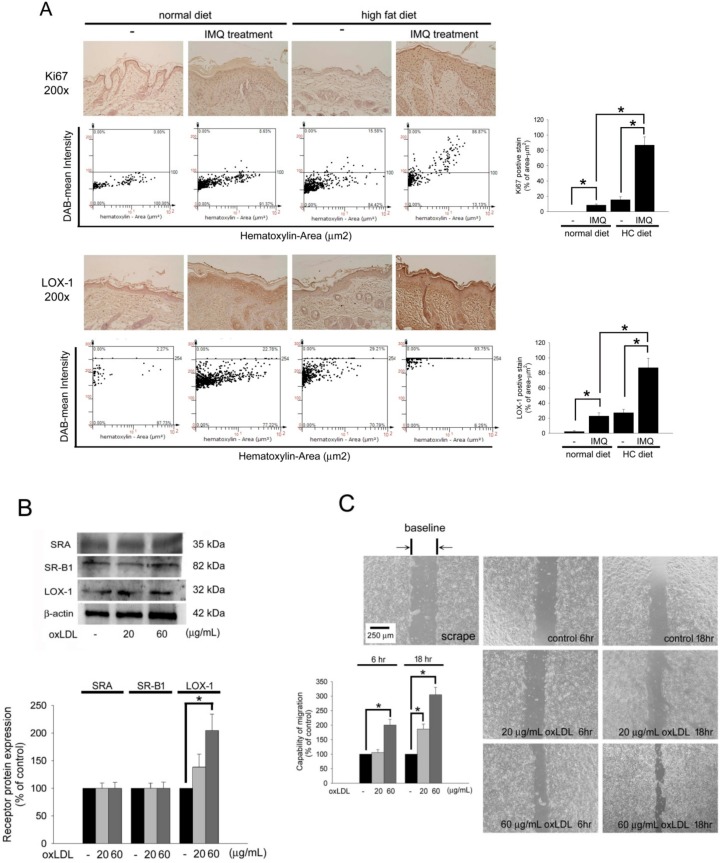
OxLDL increases keratinocytes migration and LOX-1 expression. (**A**) The skin slides were stained with anti-Ki67 or anti-LOX-1 antibodies. The images were acquired at a 200× magnification and quantified using TissueGnostics TissueFAXS & HistoFAXS System (TissueGnostics, Vienna, Austria). (**B**) Hacat cells were treated with 20 or 60 µg/mL of oxLDL, and the total protein was analyzed to assay the SR-A, SR-B1, and LOX-1 expression. β-actin was used as a loading control. The density of each band was quantified by a densitometer. (**C**) Wound healing assays for evaluating the effect of oxLDL on Hacat cell migration. Hacat cells migrating to the denuded area were counted based on the black baseline. Hacat cells were cultured with oxLDL for 24 h before wound scraping using a pipette tip. The photographs were taken 6 and 18 h after wound scraping (×40). The Hacat cells that migrated into the denuded area (double arrows indicate the denuded area) were analyzed. The magnitude of Hacat migration was evaluated by counting the migrated cells in six random clones under a high-power microscope field (×100). The results were expressed as the mean SD; * *p* < 0.05 was considered statistically significant.

In psoriasis, Ki67 will be highly expressed in migrating and proliferating keratinocyte. Therefore, immunohistochemistry was performed to assay the skin slides. In Figure 2A, Compared to non-IMQ treatment group, IMQ treatment in the normal chow diet-fed B6.129S2-Apoe^tm1Unc^/J mice slightly increased Ki67 expression on the epidermis layer. Additionally, mice fed the HC-diet only (without IMQ treatment) also had slightly induced Ki67 expression. In contrast, mice fed an HC-diet for 6 weeks plus IMQ treatment had significantly induced accumulation of Ki67 on the epidermis layer compared with in IMQ treatment plus normal chow diet-fed mice, although the thickness of the epidermis was not thicker. Since the HC diet aggravated psoriasis-like skin formation in IMQ-induced mice, major scavenger receptors, such as LOX-1, SR-B1, and SR-A for oxLDL uptake, in the skin in vivo and keratinocyte in vitro were identified. In normal chow diet-fed groups, compared to non-IMQ treatment, IMQ treatment indeed increased LOX-1 expression on the epidermis in B6.129S2-Apoe^tm1Unc^/J mice. Interestingly, the mice fed the HC alone also had increased LOX-1 expression. As expected, feeding of the HC diet plus IMQ treatment significantly induced the highest expression of LOX-1 in B6.129S2-Apoe^tm1Unc^/J mice. The performance of LOX-1 expression in the area of 1 µm^2^ was quantified using TissueGnostics TissueFAXS & HistoFAXS System. Additionally, we performed the in vitro studies. The cultured Hacat cells were stimulated with 20 or 60 µg/mL of oxLDL for 24 h, and LOX-1 but not SR-A or SR-B1 increased after stimulation (Figure 2B). The migratory functions of keratinocytes are believed to be important issues during psoriasis formation. Therefore, in vitro wound healing assays were performed. After pretreatment of cells with oxLDL for 24 h and scraping for 6 or 18 h, the migratory ability increased with 20 and 60 µg/mL oxLDL in Hacat cells (20 µg/mL oxLDL for 18 h: 200.4 ± 19.8% of the control, 60 μg/mL oxLDL for 6 h: 186.7 ± 11.7% of the control, and 60 μg/mL oxLDL for 18 h: 304.7 ± 25.8% of the control) (Figure 2C). These results indicate that oxLDL potentially increases the migration and LOX-1 expression of keratinocytes, which we predict are associated with the expression of LOX-1.

### 2.4. OxLDL Induces Keratinocyte Activity and Aggravates TNF-α Effects on Keratinocytes Mediated by LOX-1

**Figure 3:**
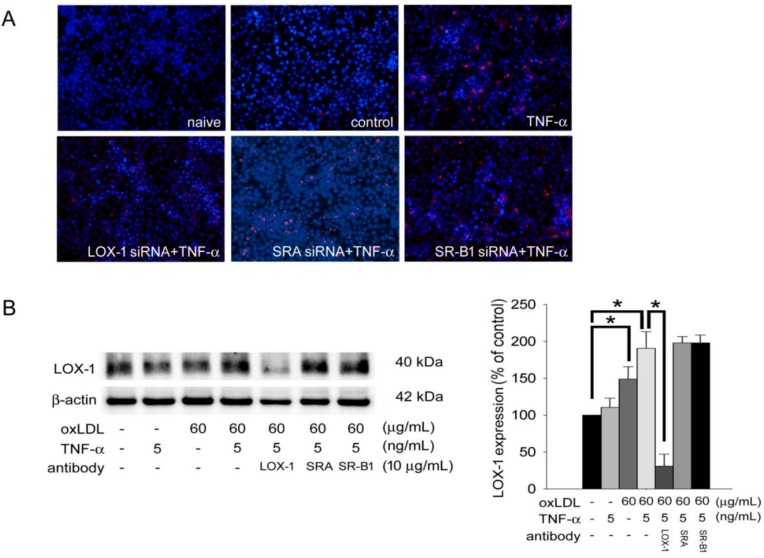

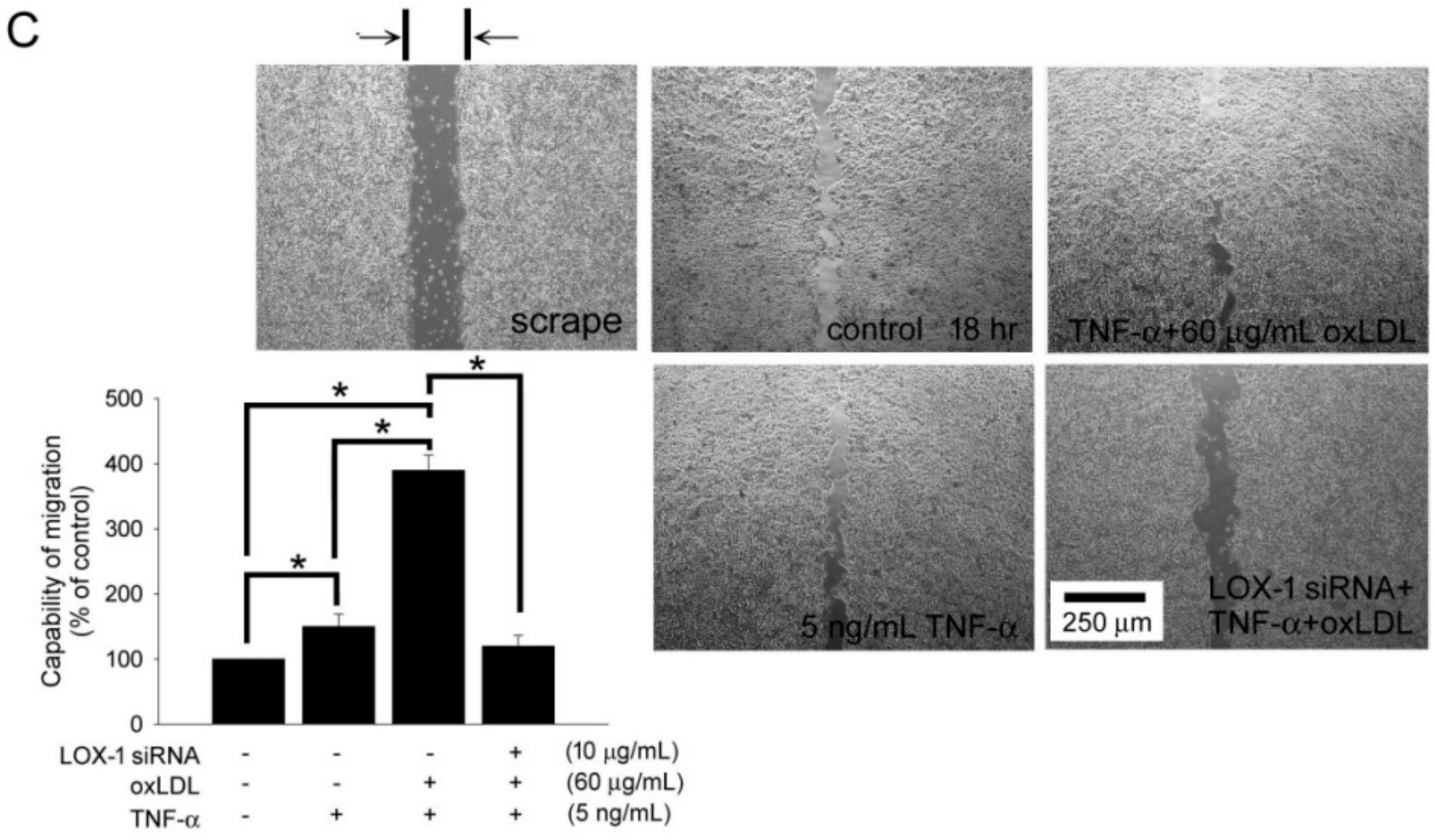
OxLDL induces keratinocytes activity and aggravates TNF-α effects to keratinocytes mediating by LOX-1. (**A**) Hacat cells were transfected with scavenger receptor shRNA for 24 h and performed with 5 ng/mL of TNF-α treatment for 24 h. After incubation of 80 µg/mL of DiI-labeled oxLDL for 24 h, the intracellular fluorescence of DiI-labeled oxLDL was observed using confocal microscopy. (**B**) Hacat cells were incubated with TNF-α, oxLDL, or oxLDL plus TNF-α for 24 h with or without preincubation of specific competitive scavenger antibodies. Total proteins were extracted and western blot analysis was performed. β-actin was used as a loading control. The density of each band was quantified by a densitometer. (**C**) Hacat cells were transfected with or without 10 μg/mL of LOX-1 siRNA for 24 h, and treatment with 5 ng/mL of TNF-α before wound scraping using a pipette tip. The photographs were taken 18 h after wound scraping (×40). The Hacat cells that migrated into the denuded area (double arrows indicate the denuded area) were analyzed. The magnitude of Hacat migration was evaluated by counting the migrated cells in six random clones under a high-power microscope field (×100). The results were expressed as the mean ± SD; * *p* < 0.05 was considered statistically significant.

Since oxLDL simultaneously increased keratinocyte migration and LOX-1 expression, we performed the DiI-labeled oxLDL uptake assay to study the significance of LOX-1 in oxLDL-stimulated keratinocyte activity. We knocked down SR-A, SR-B1, or LOX-1 gene expression using specific siRNA and then treated cells with TNF-α. Figure 3A demonstrates that compared to naïve cells, the cells in the control group took up less DiI-labeled oxLDL and the cells in the TNF-α treatment group took up significantly larger amounts of DiI-labeled oxLDL. In the cells with knocked-down LOX-1 gene expression after siRNA transfection, the red fluorescence of DiI-labeled oxLDL in the cytoplasm was significantly decreased. However, the phenomenon of DiI-labeled oxLDL uptake did not change in the SR-A or SR-B1 siRNA-transfected plus TNF-α treatment groups compared to the TNF-α treatment alone group. Furthermore, western blotting was conducted to assess the roles of scavenger receptors in TNF-α- and oxLDL-induced keratinocyte LOX-1 enhancement. Treatment with 60 µg/mL oxLDL plus 5 ng/mL TNF-α induced LOX-1 expression to reach a plateau. Pretreatment with competitive LOX-1 antibody but not the SR-A and SR-B1 antibodies reversed the increasing LOX-1 expression (Figure 3B). Finally, the wound healing assay also showed that LOX-1 mediated oxLDL-aggravated migration by TNF-α-stimulated keratinocytes (Figure 3C). These results suggest that oxLDL induces keratinocyte activity and aggravates TNF-α effects on keratinocytes by mediating LOX-1.

### 2.5. HC Diet Aggravates IL-23 Expression in IMQ-Treated B6.129S2-Apoe^tm1Unc^/J Mice, and oxLDL Induces IL-23 Expression Mediated by LOX-1 in TNF-α-Stimulated Hacat Cells

**Figure 4:**
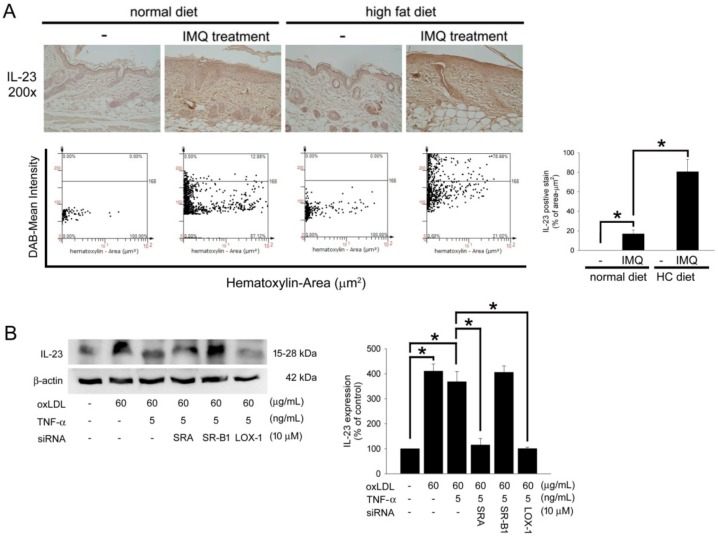
HC-diet aggravated the IL-23 expression in IMQ-treated B6.129S2-Apoe^tm1Unc^/J mice and oxLDL induced IL-23 expression mediating by LOX-1 in TNF-α-stimulated Hacat cells. (**A**) The representative photos of the mice received IMQ treatment in a normal chow diet or HC-diet groups. The skin slides were stained with anti-IL-23 antibody. The images were acquired at a 200× magnification and quantified using TissueGnostics TissueFAXS & HistoFAXS System (TissueGnostics, Vienna, Austria). The density of IL-23 expression was presented as a bar graph (right). (**B**) Hacat cells were incubated with oxLDL or oxLDL plus TNF-α for 24 h with or without preincubation of specific competitive scavenger antibodies. Total proteins were extracted and western blot analysis was performed. β-actin was used as a loading control. The density of each band was quantified by a densitometer. All results were expressed as the mean ± SD. A * *p* < 0.05 was considered statistically significant.

IL-23 is currently considered to be crucial in the pathogenesis of psoriasis. Therefore, IL-23 expression was identified in IMQ-treated, HC-diet-fed B6.129S2-Apoe^tm1Unc^/J mice. As shown in Figure 4A, the normal skin has a thin epidermis and less IL-23 presentation on the epidermis of the slide. Compared to naïve mice, the mouse skin revealed significant IL-23 expression after IMQ treatment in B6.129S2-Apoe^tm1Unc^/J mouse fed a normal chow diet. Interestingly, the HC diet may severely induce IL-23 expression in mice after IMQ treatment. Additionally, Hacat cells were stimulated with 60 µg/mL oxLDL with or without 5 ng/mL TNF-α, and western blotting showed that oxLDL induced IL-23 expression by mediating SR-A and LOX-1 (Figure 4B). These results suggest that the HC diet aggravated IL-23 expression in IMQ-treated B6.129S2-Apoe^tm1Unc^/J mice and that LOX-1-mediated oxLDL induced IL-23 expression in TNF-α-stimulated Hacat cells.

## 3. Discussion

Psoriasis is a chronic inflammatory disease characterized by patches of abnormal skin. The relationship between psoriasis and lipid metabolism has been more widely discussed. In 2009, Leslie et al. determined that IMQ, a nucleoside analog, is a topical treatment for genital and perianal warts caused by human papilloma virus. IMQ could induce psoriasis-like skin in mice, including skin inflammation, proliferation, altered differentiation of keratinocytes and increases in proinflammatory cytokines of the IL-23/IL-17 axis [20]. Xie et al. addressed the potential of IMQ-induced ApoE-deficient mice, which might be an animal model for the study of psoriasis and dyslipidemia [21]. They demonstrated that ApoE-deficient mice fed a normal chow diet with IMQ treatment for 5 days could show psoriasiform skin lesions characterized by scaling and infiltrating lesions. Additionally, IMQ-induced ApoE-deficient mice showed obviously upregulated LOX-1 expression in the epidermis. In this study, we attempted to understand the possible relationship between mechanisms of the occurrence of psoriasis and dyslipidemia, therefore, we performed the investigation using HC diet-fed B6.129S2-*Apoe^tm1Unc^*/J mice in IMQ-induced psoriasis-like animal model. Our study demonstrated similar findings and showed that, when ApoE-deficient mice were fed normal chow diets with IMQ treatment for 7 days, the PASI score and epidermal thickness were similar to those of previous studies [21]. However, when ApoE-deficient mice were fed an HC diet with IMQ treatment for 7 days, there were differences in the results. Mice fed the HC diet with IMQ treatment had more serious psoriasis. These results suggest that the HC diet aggravated psoriasis-like phenomena and epidermis hyperplasia in IMQ-treated B6.129S2-*Apoe^tm1Unc^*/J mice.

Earlier studies indicate that the low-density-lipoprotein (LDL) receptor was expressed on the epidermis [22,23]. The LDL receptor was expressed at a higher level in the psoriasis skin than in the normal skin [24,25]. Human low-density lipoprotein is the major lipid protein complex in the blood for the transport of lipids throughout the body. Human LDL changes into oxLDL by oxidative stress, inflammation and air pollutants [26]. Scavenger receptor A (SRA), SR-B1, and LOX-1 make up over 90% of the oxLDL, leading to foam cell formation and secretion of inflammatory cytokines in atherosclerosis [27]. According to a previous study, SRA receptors recognize different modifications of LDLs, including oxidized LDL, acetylation LDL, acetoacetylation LDL, and succinylation LDL in macrophages [28]. In addition to macrophages, SRA receptors were also expressed in smooth muscle cells [29] and sinusoidal endothelial cells in the liver [30]. Another receptor, SR-B1, has been shown to be a high-affinity receptor for LDL, VLDL, oxidized LDL, and acetylated LDL, which further activated the PI3K/Akt and MAPK pathways [31,32]. In the epidermis, SR-BI is the expressed cell surface receptor for high-density lipoprotein (HDL), which facilitates increased cholesterol uptake leading to barrier restoration [33]. The last receptor, LOX-1, was first identified as the main receptor for oxidized LDL (oxLDL) in endothelial cells. It is also expressed in macrophages and smooth muscle cells [34]. In the epidermis, LOX-1 was expressed in the epidermis of mice [21]. However, the relationship between oxLDL receptor and psoriasis has rarely been discussed. In our study, LOX-1 was more obviously expressed in mice fed an HC-diet with IMQ treatment than those fed a normal diet with IMQ treatment. The result implies that the HC diet may increase LOX-1 expression in the mouse epidermis. In our in vitro study, oxLDL induces keratinocyte activity and aggravates TNF-α effects on keratinocytes mediated by LOX-1.

According to a previous study, both IL-23 and IL-17 play important roles in the development of psoriatic disease [35,36]. The IL-23/IL-17 axis is involved in many different diseases, including rheumatoid arthritis, spondyloarthritis, atherosclerosis, and psoriasis [37]. IL-23 is a pro-inflammatory cytokine that is an important factor involved in the differentiation of T helper (Th17) cells [38]. Much evidence suggests that IL-23 is a main cytokine in the pathogenesis of psoriasis [39]. In a clinical study, patients with atherosclerosis had significantly increased plasma levels of IL-23, which are produced by macrophages and dendritic cells (DCs) [40,41]. In the vascular wall, oxLDL, cholesterol [42], and nicotine [43] have the capacity to induce IL-23 secretion by DCs. Therefore, it has considerable relevance between oxLDL and IL-23. In dyslipidemia, oxLDL binding to LOX-1 induces endothelial dysfunction, macrophage foam cell formation, and smooth muscle cell migration and proliferation [44]. Even though the studies demonstrate this as well [45], the relationship between the oxLDL receptor, IL-23, and LOX-1 still need to be discussed in the future.

In conclusion, we demonstrated that HC-diet aggravates psoriasis-like phenomena in IMQ-treated B6.129S2-Apoe^tm1Unc^/J mice. In vitro study, we indicate that keratinocyte uptake oxLDL through the LOX-1 but not SRA or SR-B1. Additionally, oxLDL aggravates IL-23 expression by LOX-1 in TNF-α-stimulated keratinocytes. These results have evidenced the previous clinical observation that psoriasis may be exacerbated by dyslipidemia. Ongoing study, we are analyzing the pathological changes of psoriasis after dealing with hyperlipidemia in patients who present with hyperlipidemia and psoriasis. We look forward to finding out the true roles of modified LDL in psoriasis.

## 4. Materials and Methods

### 4.1. In Vivo Animal Study

#### 4.1.1. Ethics Statement

All animals were treated according to protocols approved by the Institutional Animal Care Committee of Taipei Medical University in Mar-24, 2014 (certificate No: LAC-2014-0327). Experimental procedures and animal care conformed to the “Guide for the Care and Use of Laboratory Animals” published by the U.S. National Institutes of Health (Publication No. 85–23, revised 1996).

#### 4.1.2. Animal Grouping and Experiment Protocol

All animals were kept in microisolator cages on a 12 h day/night cycle and fed a normal murine chow diet (Scientific Diet Services, Essex, UK) or HC diet (60% of energy from fat; TestDiet 58Y1; TestDiet, St Louis, MO, USA) with water ad libitum. Twenty 6–8-week-old male B6.129S2-*Apoe^tm1Unc^*/J mice (a homozygous Apoetm1Unc mutation mouse have an elevated cholesterol level when fed a HC diet; JAX^®^, 002052, Jackson Laboratory, Bar Harbor, ME, USA) were used. The psoriasis-like skin inflammation model was modified according to the previous reference [20]. Mice were treated by daily topical application of a dose of 62.5 mg/cm^2^ 5% IMQ cream on the shaved back for 7 consecutive days. All animals were randomly divided into four groups with 5 mice in each group (Figure 1A): group 1 (naïve control) mice were fed a normal chow diet; group 2 mice were fed a normal chow diet and received IMQ treatment at the beginning of experimental week 6; group 3 mice were fed the HC diet; and group 4 mice were fed the HC diet and received IMQ treatment at the beginning of experimental week 6. At the end of the experiment (end of week 6/day 42), the mice were sacrificed, and the treated skin was removed. To evaluate the severity of the inflammation of the shaved skin, the Psoriasis Area and Severity Index (PASI) method was used. Each of four symptoms (erythema, scaling, thickness, and cumulative scores) was scored separately according to the following scale: 0 (not present), 1 (mild), 2 (moderate), or 3 (severe), summed up and an integration bar was drawn for each group.

#### 4.1.3. Biochemical Measurements

Blood samples for biochemical measurements were collected from each animal before starting the experiment, at the end of 6 weeks, and at sacrifice. Samples were collected from the mandibular artery into sodium citrate-containing tubes and separated by centrifugation. Serum BUN, ALT, AST, total-cholesterol, and TG were measured using the SPOTCHEMTM automatic dry chemistry system (SP-4410; Arkray, Tokyo, Japan).

#### 4.1.4. Immunohistochemistry

The animals were sacrificed at the end of week 6, and the back skin was harvested, gently dissected, rinsed with ice-cold phosphate buffered saline, immersion-fixed with 4% buffered paraformaldehyde, paraffin-embedded, and 5-µm-thick paraffin-embedded cross-sections of mouse skin were stained. Immunohistochemical staining used anti-LOX-1 (Santa Cruz, Dallas, TX, USA), anti-Ki67 (Abcam, San Francisco, CA, USA), and host anti-IL23 (Novusbio, Littleton, CO, USA) antibodies. The slides were observed via TissueGnostics TissueFAXS & HistoFAXS System (TissueGnostics, Vienna, Austria).

### 4.2. In Vitro Study

#### 4.2.1. Cell Culture

The human immortalized keratinocyte cell line, HaCaT cells, were cultured in Dulbecco’s modified Eagle’s medium supplemented with 2 mm glutamine, antibiotics (100 U/mL of penicillin A and 100 U/mL of streptomycin) and 10% fetal bovine serum (Gibco/BRL, Grand Island, NY, USA) and maintained in a 37 °C humidified incubator containing 5% CO_2_. Cells were serially passaged at 70–80% confluence. When performing experiments, the HaCaT cells were grown to 90% confluence.

#### 4.2.2. Western Blotting Analysis

Total cell lysates were extracted from keratinocytes. Proteins were separated by SDS-PAGE and transferred to a PVDF membrane. The membranes were probed using anti-Ki67 (Santa Cruz, CA, USA), anti-LOX-1 (Santa Cruz, Dallas, TX, USA), anti-scavenger receptor A (SRA; Santa Cruz, CA, USA), anti-SR-B1 (Santa Cruz, Dallas, TX, USA), and anti-IL23 (Novusbio, Littleton, CO, USA) antibodies. Mouse anti-β-actin (Labvision/NeoMarkers, Fremont, CA, USA) antibody was used as a loading control. The proteins were visualized using an enhanced chemiluminescence (ECL) detection kit (Amersham Biosciences, Waltham, MA, USA) and quantified using a densitometry.

#### 4.2.3. Wound Healing Assay

The migratory capability of keratinocytes is associated with the occurrence of psoriasis. Therefore, a wound healing assay was used. Keratinocytes were cultured in a 12-well plate. The confluent cells (approximately 2 × 10^5^ cells/well) were wounded by scraping with a 200 µL pipette tip, which denuded a strip of monolayer that was 300 µm in diameter. The cells were supplied with medium containing 5% fetal bovine serum, and the rate of wound closure was observed after 6 or 18 h. The distance of the gap was measured under the 4× objective of a light microscope (Olympus IX71, Tokyo, Japan), monitored with a CCD camera (Macro FIRE 2.3A), and captured with a video graphic system (Picture Frame Application 2.3 software).

#### 4.2.4. DiI-Labeled oxLDL Uptake Assay

Human LDL (density: 1.019–1.063 g/mL) was isolated by sequential ultracentrifugation of fasting plasma samples from healthy adult males. Native LDL was oxidized as described in our previous report [46]. The oxLDL was labeled with 1,1′-dioctadecyl-3,3,3′,3′-tetramethyl-indocarbocy-anine perchlorate (DiI) as described previously [47]. To examine the cellular uptake of oxLDL, Keratinocytes were seated on culture slides and incubated for 4 h in culture medium containing 80 µg/mL of DiI-labeled oxLDL. At the end of the treatment, the cells were washed with PBS, mounted on cover slips, and examined by confocal microscopy.

#### 4.2.5. Statistical Analysis

Results were expressed as the mean ± SEM. Data were analyzed using ANOVA followed by the Dunnett’s test. A *p* value less than 0.05 was considered statistically significant.

## Figures and Tables

**Table 1:** Plasma biochemical characteristics (*n* = 5) in experimental animals.

Biochemical Characteristics	Weeks	Normal Diet		HC Diet	
		IMQ Treatment		IMQ Treatment	
BUN (mg/dL)	0	28.3 ± 6.0	26.3 ± 6.5	27.2 ± 7.8	26.9 ± 5.2
	6	26.2 ± 8.0	27.4 ± 8.1	26.4 ± 7.4	28.4 ± 7.2
Creatinine (mg/dL)	0	0.8 ± 0.2	0.7 ± 0.5	0.9 ± 0.4	0.8 ± 0.3
	6	0.6 ± 0.1	0.8 ± 0.2	0.7 ± 0.5	0.7 ± 0.4
AST (IU/L)	0	25.7 ± 3.8	25.6 ± 2.8	26.8 ± 3.4	264 ± 4.2
	6	26.7 ± 3.2	27.4 ± 3.6	27.4 ± 2.8	28.3 ± 3.4
ALT (IU/L)	0	35.5 ± 9.1	34.4 ± 12.3	34.6 ± 8.2	35.3 ± 7.6
	6	34.8 ± 6.4	36.8 ± 10.2	34.7 ± 6.4	34.8 ± 5.2
Total-cholesterol (mg/dL)	0	28.6 ± 6.2	25.2 ± 3.2	26.4 ± 7.0	27.3 ± 5.8
	6	29.5 ± 7.3	27.6 ± 4.8	1213.5 ± 174.2 *	1047.0 ± 198.2 *
TG (mg/dL)	0	16.7 ± 8.3	18.2 ± 6.4	20.4 ± 2.8	19.4 ± 5.8
	6	14.8 ± 7.2	17.2 ± 5.3	92.0 ± 18.4 *	94.0 ± 17.7 *

HC diet, high-cholesterol diet; BUN, blood urea nitrogen; ALT, alanine aminotransferase; AST, aspartate aminotransferase; TG, triglyceride; IMQ, imiquimod. Values are mean ± SD. * *p* < 0.05 compared with 0 week of the same group.

## Abbreviations

IMQ	imiquimod
TNF-α	tumor necrosis factor alpha
oxLDL	low-density lipoprotein
LOX-1	lectin-like oxidized low-density lipoprotein LDL receptor-1
BUN	serum blood urea nitrogen
ALT	alanine aminotransferase
AST	aspartate aminotransferase
DiI	1,1′-dioctadecyl-3,3,3′,3′-tetramethyl-indocarbocyanine perchlorate
IL	interleukin
